# Integrated analyses of single-cell transcriptomics identify metastasis-associated myeloid subpopulations in breast cancer lung metastasis

**DOI:** 10.3389/fimmu.2023.1180402

**Published:** 2023-07-07

**Authors:** Zhen Huang, Dawei Bu, Nan Yang, Wenwen Huang, Liyin Zhang, Xiaoxue Li, Bi-Sen Ding

**Affiliations:** Key Laboratory of Birth Defects and Related Diseases of Women and Children of MOE, State Key Laboratory of Biotherapy, West China Second University Hospital, Sichuan University, Chengdu, China

**Keywords:** breast cancer, lung metastasis, single-cell transcriptome, myeloid cells, immunity

## Abstract

Lung metastasis of breast cancer is closely associated with patient morbidity and mortality, which correlates with myeloid cells in the lung microenvironment. However, the heterogeneity and specificity of metastasis-associated myeloid cells have not been fully established in lung metastasis. Here, by integrating and analyzing single-cell transcriptomics, we found that myeloid subpopulations (*Tppp3* ^+^ monocytes, *Isg15* ^+^ macrophages, *Ifit3* ^+^ neutrophils, and *Il12b* ^+^ DCs) play critical roles in the formation and development of the metastatic niche. Gene enrichment analyses indicate that several tumor-promoting pathways should be responsible for the process, including angiogenesis (*Anxa1* and *Anxa2* by *Tppp3* ^+^ monocytes), immunosuppression (*Isg15* and *Cxcl10* by *Isg15* ^+^ macrophages; *Il12b* and *Ccl22* by *Il12b* ^+^ DCs), and tumor growth and metastasis (*Isg15* and *Isg20* by *Ifit3* ^+^ neutrophils). Furthermore, we have validated these subpopulations in lung microenvironment of MMTV-PyVT transgenic mice and verified their association with poor progression of human breast cancer. Also, our results elucidated a crosstalk network among four myeloid subpopulations by cell-cell communication analysis. This study, therefore, highlights the crucial role of myeloid cells in lung metastasis and provides insights into underlying molecular mechanisms, which pave the way for therapeutic interventions in breast cancer metastasis to lung.

## Introduction

Breast cancer is the most common cancer worldwide and spreads preferentially to the lung, brain, bone, and liver (1, 2). In particular, lung metastases can occur in initial breast cancer diagnosis and have a significant impact on patient morbidity and mortality (3). In the past, myeloid cells of granulocytic and monocytic lineages, components of innate immunity, have been reported to facilitate cancer metastasis to lung, including monocytes, macrophages, neutrophils, and dendritic cells (DCs) (4). But, the underlying molecular mechanisms of that have not been fully explained. The heterogeneity and specificity of innate myeloid populations associated with breast cancer lung metastasis have been also rarely established.

The technology of single-cell sequencing has greatly improved the resolution of vision in cancer research, making it possible to have insight into the state of individual cells. Particularly, the cellular heterogeneity can be interpreted more clearly, including genome, transcriptome, proteome, and epigenomics (5). With this technology, new subtypes of leukocytes have been found and identified (6). Recent studies involved in innate immune cells from tumors and adjacent lung tissue of lung cancer patients identified immune subtypes and differentially regulated genes (7, 8). Previously, two single-cell transcriptomics of breast cancer lung metastasis have been reported in mouse tumor models (9, 10). These analyses provide new molecular insights into underlying mechanisms and contribute to the design of therapeutic strategies. But there is no systematic and in-depth exploration for characteristics of tumor-associated myeloid cells in breast cancer metastasis to lung. In this study, we collected and integrated scRNA-seq (single-cell transcriptome sequencing) datasets from GEO (Gene Expression Omnibus). By differential analysis of myeloid cell populations in normal lung and metastatic lung with different stages, we explored the heterogeneity and specificity of innate myeloid cells in tumor-metastasis lung microenvironment and interactions among different myeloid subpopulations.

## Materials and methods

### The lung-metastasis model of mouse breast cancer

FVB/N-Tg (MMTV-PyVT) 634Mul/J (MMTV-PyVT) mice were purchased from the Jackson Laboratory (11). Hemizygous MMTV-PyVT females develop palpable mammary tumors which metastasize to the lung. Female mice develop palpable mammary tumors with a mean latency of 60 days of age. Tumor-bearing females have 80-90% incidence of lung metastasis at 100 days of age. Mice at 130 days of age are sacrificed and lung tissues are collected for analysis. All mice were housed under specific-pathogen-free conditions and fed autoclaved food at the Experimental Center of West China Second University Hospital. The protocols for animal experiments were approved by the Laboratory Animal Ethics Committee of West China Second University Hospital, Sichuan University.

### Immunofluorescence and pathological analyses

The fresh tissues were collected after euthanasia of MMTV-PyVT mice and embedded with opti-mum cutting temperature compound (O.C.T. Compound), frozen at -80°C, and cut into sections for immunofluorescence on a freezing microtome (Leica). Then, sections were incubated in blocking solution (0.4% Triton X-100 and 10% donkey serum in phosphate buffer saline) for 30 min at room temperature, followed by incubating in blocking solution containing primary antibodies overnight at 4°C in the dark. Primary antibodies used were rabbit anti-LY6C (1:200, ab54223, Abcam), rabbit anti-F4/80 (1:200, ab300421, Abcam), rabbit anti-MPO (1:200, 22225-1-AP, Proteintech), armenian hamster anti-CD11c (1:200, MA11C5, ThermoFisher), mouse anti-TPPP3 (1:200, 15057-1-AP, Proteintech), mouse anti-ISG15 (1:50, sc-166755, Santa cruz), mouse anti-IFIT3 (1:50, sc-393512, Santa cruz), and mouse anti-IL12B (1:50, sc-365389, Santa cruz). Sections were washed in wash buffer three times and then incubated in a blocking solution containing secondary antibodies for two hours at room temperature, followed by incubating in 4’,6-diamidino-2-phenylindole (DAPI) (1:5,000, 10236276001, Roche) for 15 min. The paraffin sections of the lung from MMTV-PyVT mice were stained with H&E and analyzed for pathological changes.

### Acquisition and collection of single-cell RNA sequencing datasets

Breast cancer datasets containing mouse myeloid cells used in this study include GSE109281 and GSE129578 from GEO. ScRNA-seq data of GSE109281 was generated from CD45^+^ mouse immune cells of blood, lungs, and primary tumors in normal or tumor-bearing mice through the 10X Genomics platform (10). The cell data without additional treatment was used in integrated analysis. ScRNA-seq data of GSE129578 was generated from CD45^+^ mouse immune cells of lungs and primary tumors in normal or tumor-bearing mice through the 10X Genomics platform (9). The samples of tumor-bearing mice are dissected from a patient-derived xenograft (PDX) mouse model of metastatic triple-negative breast cancer in immunodeficient mice lacking adaptive immunity. The data contains cells from lungs with different metastatic stages. The diameter of tumors in the lung was used as a proxy for metastatic progression as follows: early-stage lung (< 0.5cm diameter), mid-stage lung (~1.5cm diameter), and late-stage lung (> 2.5cm diameter). We used these labels of cells for the definition of metastatic stages in our studies. Human breast cancer dataset used in this study is GSE114727 from GEO. ScRNA-seq data of GSE114727 was generated from CD45^+^ human immune cells of breast cancer patients through the inDrop platform (12). The cell data from peripheral blood, primary tumor, and matched normal breast tissue was used in the analysis.

### Integration and analysis of single-cell transcriptomes

All scRNA-seq matrix counts used in this study are obtained from GEO. R software (version: 4.1.0) and the “Seurat” package were used for scRNA-seq integration, analysis, and visualization (13). Before the integration, several processes are performed dividually for each dataset, including quality control and cell selection, normalizing the data, and identification of highly variable features. Mouse data was integrated to better understand cellular diversity in different tissues according to integration methods of the “Seurat” package. The cross-dataset pairs of cells in a matched biological state are identified. And technical differences between datasets are also corrected. The two mouse datasets are integrated together for subsequent analysis. Human data from different patients and tissues are also integrated for correcting sample differences by the same methods. Then, data is analyzed according to the standard processing workflow of the “Seurat”, including scaling the data, linear (PCA) and non-linear (UMAP) dimensional reduction, and clustering the cells. The appropriate resolution for UMAP visualization was determined using the “Clustree” package (14). After that, differentially expressed features are found and cell type identity to clusters is assigned. Visualizations of data analysis in this study are also performed by the “Seurat”, including scatter plots, violin plots, dot plots, volcano plots, and heatmap plots. Visualization of cluster distribution in different resolutions is performed by the “Clustree”.

### Identification of cell types

Cell types of clusters are identified by the “Singler” package, based on RNA sequencing data of human or mouse immune and stroma cells (15). Biomarkers of cell types previously reported in the original articles are used as supplements and references (9, 10, 12). The following cell types and representative marker genes are found in mouse data: monocyte (*Ccr2*, *Csf1r*), neutrophil (*S100a8*, *S100a9*), DC (*Siglech*, *Irf8*), macrophage (*C1qa*, *C1qb*), basophil (*Cpa3*, *Ms4a2*), fibroblast (*Col1a1*, *Col3a1*), and endothelial cell (*Pecam1*, *Kdr*). The following cell types and representative marker genes are found in human data: T cell (*CD3E*, *CD3D*), NKT cell (*NKG7*, *GNLY*), NK cell (*NCAM1*, *NCR1*), B cell (*CD19*, *MS4A1*), neutrophil (*S100A8*, *S100A9*), monocyte (*FCGR3A*, *CD14*), DC (*CST3*, *CD1C*), macrophage (*CD68*, *CSF1R*), mast cell (*TPSB2*, *KIT*), epithelial cell (*KRT18*, *KRT19*), fibroblast (*MGP*, *DCN*), vascular endothelial cell (*SPARCL1*, *IFI27*), and lymphatic endothelial cell (*TFF3*, *FABP4*).

### Gene enrichment analysis

The differentially expressed genes of clusters are obtained from analysis by the “Seurat”. Lists of genes with up-regulated or down-regulated expression in clusters are imported into the “David” online tool (https://david.ncifcrf.gov/summary.jsp), which is the database for annotation, visualization, and integrated discovery (DAVID) providing a comprehensive set of functional annotation tools for investigators to understand the biological meaning behind large lists of genes (16). Gene enrichment analysis of Gene ontology (GO) biological process is performed with the gene lists.

### Survival analysis of human breast cancer

The log-rank (Mantel-Cox) test was used to compare the groups in the Kaplan-Meier graphs. Kaplan-Meier curves of human breast invasive carcinoma (BRCA) were analyzed using TIMER2.0 (17), a comprehensive resource for systematical analysis of immune infiltrates across diverse cancer types. The human data used for analysis were from the TCGA (The Cancer Genome Atlas). The immune infiltrates and signature expression were combined to explore the clinical relevance of human breast cancer. The cut-off value of high or low is 50% in all analyses.

### Cell-cell communication analysis

The original matrix counts are extracted from the integrated data by the “Seurat”. And mouse gene names are replaced by homologous human genes in the counts. Cell-cell communication analysis is performed with the “Python” (version: 3.6) by the “CellPhoneDB” tool, a publicly available repository of curated receptors, ligands, and their interactions in human (18). For the subsequent visualizations, heatmap plots, dot plots, and shell plots are produced in the R software. The illustrations in this study were created with icons from BioRender.com.

## Results

### Integration and analysis of mouse scRNA-seq data of breast cancer lung metastasis

To explore the differences in immune microenvironment between normal lung and metastatic lung of breast cancer, we collected two scRNA-seq datasets (GSE109281 and GSE129578) about breast cancer lung metastasis, which are generated from mouse tumor models, to better understand the molecular mechanism at the individual cell level. The datasets were both produced based on the 10X Genomics platform (9, 10). For a more complete and reliable analysis, we integrated two mouse datasets and corrected technical differences between different datasets by the “Seurat” package (13). Cells of the two datasets are equally distributed in the UMAP plots (**Supplementary Figures 1A, B**).

**Figure 1:**
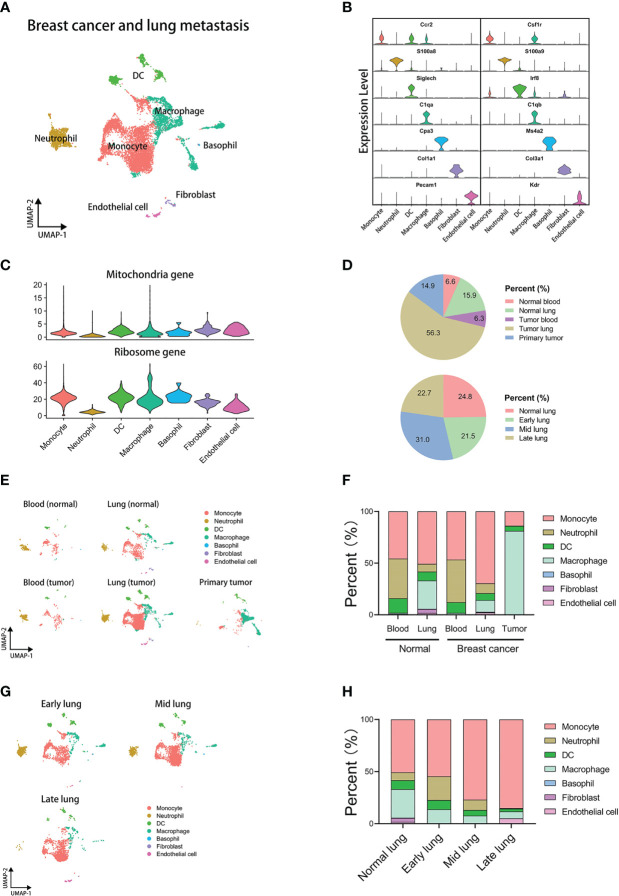
Integrated analysis of mouse single-cell transcriptomics of breast cancer lung metastasis. **(A)** UMAP plot of 11171 annotated cells in integrated datasets of breast cancer lung metastasis. **(B)** Violin plots displaying marker genes of identified subsets. **(C)** Violin plots displaying mitochondria and ribosome genes of subsets. **(D)** The pie plots comparing cell proportions of different tissues or lung metastasis stages. **(E)** UMAP clustering of subsets in different tissues. **(F)** The histogram comparing cell proportions of subsets in different tissues. **(G)** UMAP clustering of subsets in different stages of lung metastasis. **(H)** The histogram comparing cell proportions of subsets in different stages of lung metastasis.

Through the standard workflow of single-cell analysis, we identified 7 cell types in the integrated data, including monocyte, neutrophil, DC, macrophage, basophil, fibroblast, and endothelial cell (**Figures 1A, B**). The analysis of the gene components indicated that these cells have low expression of mitochondria genes but not ribosome genes (**Figure 1C**). The integrated data of breast cancer lung metastasis contains different tissues and pathological groups, and proportions of all groups are displayed by the pie plots (**Figure 1D**). The pie plot at the top displays cell precent of different tissues using integrated data of GSE109281 and GSE129578. The pie plot below displays cell precent of different lung-metastasis stages using GSE129578. The results show that the group of tumor-metastasis lung has the most cells in analysis. Furthermore, we employed the cells of GSE129578 for further grouping, which are from xenograft models of human breast cancer cells in immunodeficient mice. The cells generated from metastatic lung are from different metastasis stages and labeled by normal lung, early-stage lung, middle-stage lung, and late-stage lung, which contributes to better dissecting the tumor-reprogrammed lung microenvironment in subsequent analysis. Next, we explored the cell distribution and proportions of all cell types across different tissues (**Figures 1E, F**). The results show that innate myeloid cells (monocytes, neutrophils, DCs, and macrophages) make up almost all the cells in analysis. And there is no difference in the blood cell proportions between normal and tumor groups, but cell proportions of lung between the two groups are obviously distinct. To explore the difference in cell percent in different lung-metastasis stages of breast cancer, we performed a comparation analysis in lung-metastasis groups (**Figures 1G, H**). The results show that the proportions of monocytes have an increasing trend in the process of breast cancer metastasis to lung, and the proportions of macrophages and DCs have a decreasing trend. Moreover, neutrophil subset has the highest proportions in the early-metastasis stage. The above results indicate that myeloid cells highly correlate with lung metastasis of breast cancer.

### *Tppp3* ^+^ monocytes are associated with lung metastasis through angiogenesis

To know which subpopulations of myeloid cells are associated with lung metastasis, we extracted four subsets of myeloid cells from the integrated data for subsequent analysis respectively. The analyzed cells of the lung metastasis group are the cells that have been divided into early, middle, and late stages previously.

**Figure 2:**
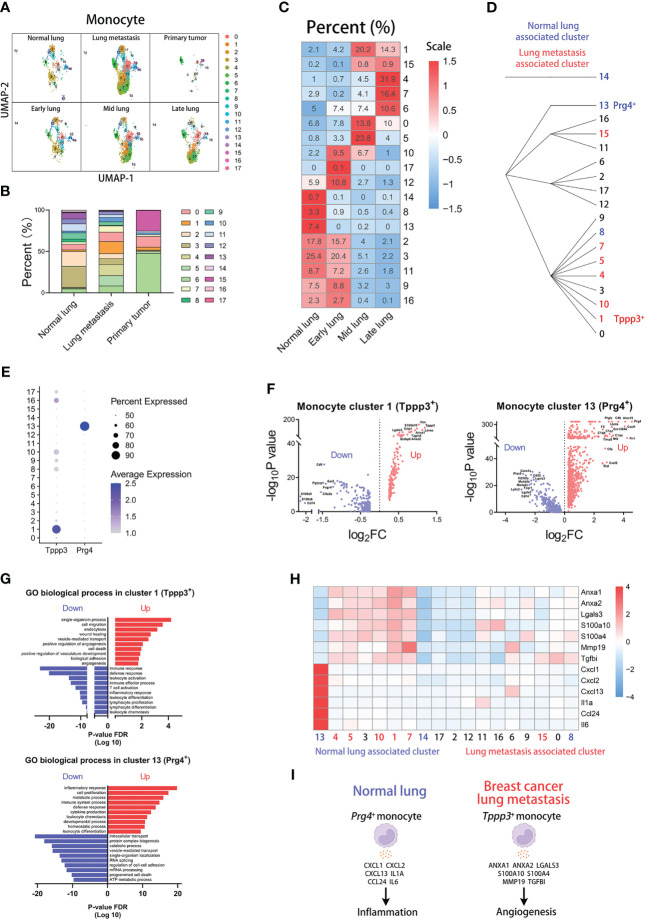
*Tppp3* ^+^ monocytes correlate with lung metastasis of breast cancer. **(A)** UMAP clustering of monocytes in different tissues and lung metastasis stages. **(B)** The histogram comparing the percent of monocyte clusters in different tissues. **(C)** The heatmap displaying the percent of monocyte clusters in different lung metastasis stages. **(D)** Hierarchical clustering of monocyte clusters. **(E)** The dot plot of *Tppp3* and *Prg4* across monocyte clusters. **(F)** The volcano plots of differential gene expression of *Tppp3* ^+^ and *Prg4* ^+^ monocytes. **(G)** The enrichment analysis of GO biological process in *Tppp3* ^+^ and *Prg4* ^+^ monocytes. **(H)** The heatmap displaying the differential expression of genes in monocyte clusters. **(I)** The schematic diagram displaying characteristics of *Prg4* ^+^ monocyte in normal lung and *Tppp3* ^+^ monocyte in breast cancer lung metastasis.

Firstly, we reanalyzed the monocyte subset and selected the appropriate resolution of dimensional reduction by the “Clustree” package (**Supplementary Figure 2A**) (14). Monocytes were divided into 17 clusters at the resolution of 1.8. The top marker genes of all clusters are shown in the heatmap (**Supplementary Figure 2F**). The analyses of mitochondria and ribosome genes are shown in the violin plot (**Supplementary Figure 6E**). According to differentially expressed genes and clustering route, we found seven monocyte subpopulations: *Ly6c2* ^+^ *Chil3* ^+^ *Plac8* ^+^ monocytes, *Pglyrp1* ^+^ *Fcgr4* ^+^ *Cd300e* ^+^ monocytes, *Gm42418* ^+^ *Malat1* ^+^ *AY036118* ^+^ monocytes, *S100a9* ^+^ *S100a8* ^+^ *Ctla2a* ^+^ monocytes, *Cd209a* ^+^ *Cd74* ^+^ *Cd7* ^+^ monocytes, *Prg4* ^+^ *Fn1* ^+^ *Alox15* ^+^ monocytes, and *Alas2* ^+^ *Bpgm* ^+^ *Snca* ^+^ monocytes (**Supplementary Figures 2B, C**). To further explore which monocyte subpopulations correlate with tumor progress and lung metastasis, we compared distribution and proportion of 17 clusters in different groups. There are only slight differences in blood between normal and tumor groups (**Supplementary Figures 2D, E**). However, in the comparison analysis between normal lung and metastatic lung, some clusters showed a wide variation in percent (**Figures 2A, B**). Lung metastasis group has higher proportions of cluster 1, 4, 5, 7, and 10 than normal lung group. However, normal lung group has higher proportions of cluster 2, 3, 11, and 13 than lung metastasis group. Then, we sought to discover the clusters which have an evident proportion trend in the process of breast cancer metastasis to lung. The heatmap and histogram display proportions of each cluster in different lung-metastasis stages (**Figure 2C** and **Supplementary Figure 6A**). The results indicate that cluster 1, 4, 5, 7, 10, and 15 with increasing proportions during metastatic stages are associated with lung metastasis, especially cluster 1 (*Tppp3* ^+^ monocytes). And cluster 8, 13, and 14 with decreasing proportions during metastatic stages are associated with normal lung, especially cluster 13 (*Prg4* ^+^ monocytes). The hierarchical clustering generated from the “Clustree” indicates that the most of metastasis-associated clusters come from the same population (**Figure 2D**).

For further exploring characteristics of the clusters associated with lung metastasis or normal lung, we analyzed differentially expressed genes of cluster 1 or 13. The dot plot indicates that *Tppp3* is mainly expressed in cluster 1 and *Prg4* is mainly expressed in cluster 13 (**Figure 2E**). The volcano plot displays up-regulated and down-regulated genes of cluster 1 or 13 compared with other clusters, showing that cluster 1 has the highest up-regulated expression of *Tppp3* and cluster 13 has the highest up-regulated expression of *Prg4* (**Figure 2F**). Then, we performed gene enrichment analysis in differentially expressed genes of cluster 1 or 13 (**Figure 2G**). The results of the GO biological process show that cluster 1 (*Tppp3* ^+^ monocytes), a lung metastasis-associated cluster, up-regulates the “angiogenesis” pathway. And cluster 13 (*Prg4* ^+^ monocytes), a normal lung-associated cluster, up-regulates the “inflammatory response” pathway. Next, we selected some genes associated with angiogenesis or inflammation for analysis, which have high fold-change in cluster 1 or 13 (**Figure 2H**). The heatmap indicates that these angiogenesis genes are abundantly expressed in cluster 1, including *Anxa1*, *Anxa2*, *Lgals3*, *S100a10*, *S100a4*, *Mmp19*, and *Tgfbi* (19–24). And the inflammation genes are highly expressed in cluster 13, including *Cxcl1*, *Cxcl2*, *Cxcl13*, *Il1a*, *Ccl24*, and *Il6*. In a word, we found that *Tppp3* ^+^ monocytes with high expression of angiogenesis genes are associated with lung metastasis, and *Prg4* ^+^ monocytes with high expression of inflammation genes are associated with normal lung (**Figure 2I**).

### *Isg15* ^+^ macrophages are associated with lung metastasis through immunosuppression

**Figure 3:**
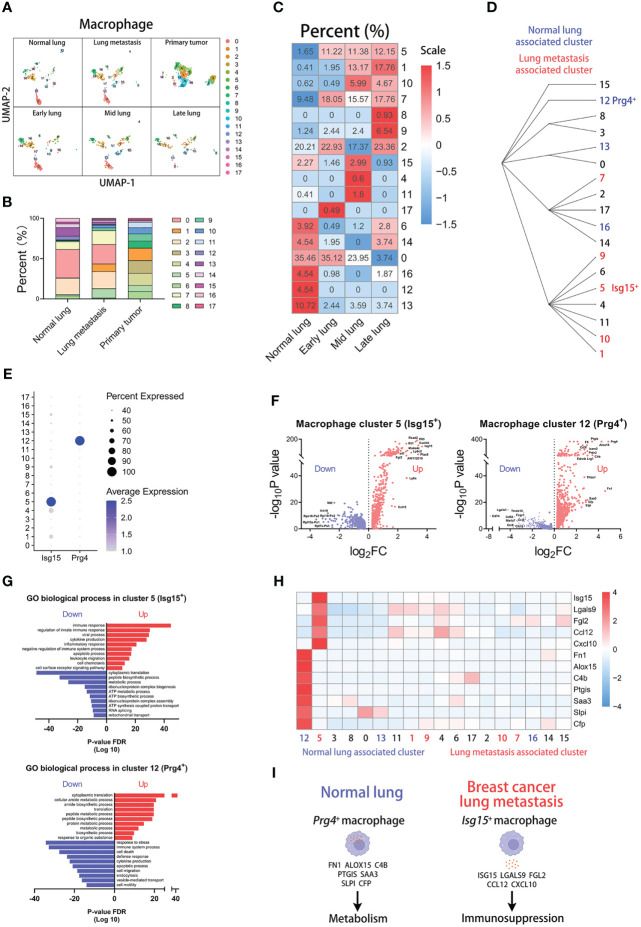
*Isg15* ^+^ macrophages correlate with lung metastasis of breast cancer. **(A)** UMAP clustering of macrophages in different tissues and lung metastasis stages. **(B)** The histogram comparing the percent of macrophage clusters in different tissues. **(C)** The heatmap displaying the percent of macrophage clusters in different lung metastasis stages. **(D)** Hierarchical clustering of macrophage clusters. **(E)** The dot plot of *Isg15* and *Prg4* across macrophage clusters. **(F)** The volcano plots of differential gene expression of *Isg15* ^+^ and *Prg4* ^+^ macrophages. **(G)** The enrichment analysis of GO biological process in *Isg15* ^+^ and *Prg4* ^+^ macrophages. **(H)** The heatmap displaying the differential expression of genes in macrophage clusters. **(I)** The schematic diagram displaying characteristics of *Prg4* ^+^ macrophage in normal lung and *Isg15* ^+^ macrophage in breast cancer lung metastasis.

We next investigated the heterogeneity of macrophages using the same parameters and methods as above. Macrophages are divided into 17 clusters at the resolution of 1.8 (**Supplementary Figure 3A**). The heatmap displays the top marker genes of all clusters (**Supplementary Figure 3F**). And the violin plot compares the fraction of mitochondria and ribosome genes among all clusters (**Supplementary Figure 6F**). In particular, some clusters share similar signatures. Through this, we found six subpopulations of macrophages: *Apoe* ^+^ *Ms4a7* ^+^ *Ly6e* ^+^ macrophages, *Mgl2* ^+^ *Tnip3* ^+^ *Cd74* ^+^ macrophages, *Ear1* ^+^ *Ear2* ^+^ *Ear10* ^+^ macrophages, *Gm9844* ^+^ *Cryab* ^+^ *Eef1g* ^+^ macrophages, *Prg4* ^+^ *Fn1* ^+^ *Alox15* ^+^ macrophages, *Birc5* ^+^ *Tuba1b* ^+^ *Top2a* ^+^ macrophages (**Supplementary Figures 3B, C**). In the analysis of cluster proportions across groups, there is no sense in the analysis of blood macrophages for too few cells in the blood group (**Supplementary Figures 3D, E**). However, the high variations of macrophage cluster proportions appear in the comparison of other groups (**Figures 3A, B**), particularly normal lung and other lung-metastasis stages (**Figure 3C** and **Supplementary Figure 6B**). Cluster 1, 5, and 7 have higher proportions in lung metastasis group than that in normal lung group, and cluster 12, 13, 14, and 16 have higher proportions in normal lung group than that in lung metastasis group. Moreover, the results show that cluster 1, 5, 7, 9, and 10 with increasing proportions during metastatic stages are associated with lung metastasis, especially cluster 5 (*Isg15* ^+^ macrophages). And cluster 12, 13, and 16 with decreasing proportions during metastatic stages are associated with normal lung, especially cluster 12 (*Prg4* ^+^ macrophages) and cluster 13 (high expression of mitochondria genes). The hierarchical clustering described their clustering relationships (**Figure 3D**).

Then, we explored the characteristics of the two clusters. We found that *Isg15* is mainly expressed in cluster 5 and *Prg4* is mainly expressed in cluster 12 (**Figure 3E**). The volcano plot shows the differentially expressed genes of cluster 5 or 12 (**Figure 3F**). Furthermore, the gene enrichment analyses for the GO biological process indicate that the metastasis-associated *Isg15* ^+^ macrophages highly express the genes related to immune response, and normal lung-associated *Prg4* ^+^ macrophages have high expression in the genes related to the metabolic process (**Figure 3G**). We next focused on the top-up-regulated genes in the clusters. And our results indicate that cluster 5 has a high expression of immunosuppression genes, including *Isg15*, *Lgals9*, *Fgl2*, *Ccl12*, and *Cxcl10* (25–29). Some genes contributing to metabolism are highly expressed in cluster 12, including *Fn1*, *Alox15*, *C4b*, *Ptgis*, *Saa3*, *Slpi*, and *Cfp* (**Figure 3H**) (30). The above results show that *Isg15* ^+^ macrophages with high expression of immunosuppression genes are associated with lung metastasis, and *Prg4* ^+^ macrophages with high expression of metabolic genes are associated with normal lung (**Figure 3I**).

### *Ifit3* ^+^ neutrophils are associated with lung metastasis through tumor growth and metastasis

**Figure 4:**
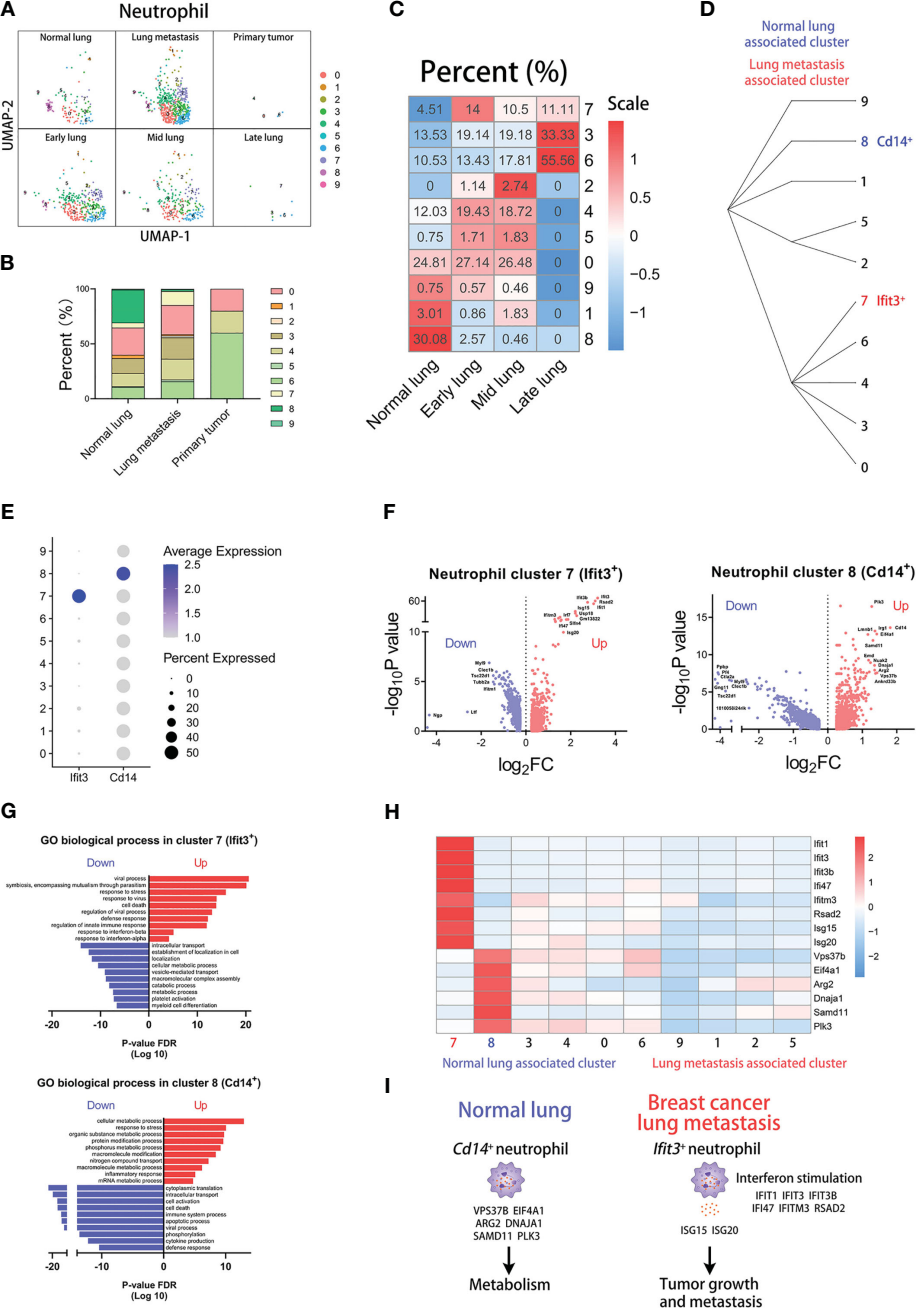
*Ifit3* ^+^ neutrophils correlate with lung metastasis of breast cancer. **(A)** UMAP clustering of neutrophils in different tissues and lung metastasis stages. **(B)** The histogram comparing the percent of neutrophil clusters in different tissues. **(C)** The heatmap displaying the percent of neutrophil clusters in different lung metastasis stages. **(D)** Hierarchical clustering of neutrophil clusters. **(E)** The dot plot of *Ifit3* and *Cd14* across neutrophil clusters. **(F)** The volcano plots of differential gene expression of *Ifit3* ^+^ and *Cd14* ^+^ neutrophils. **(G)** The enrichment analysis of GO biological process in *Ifit3* ^+^ and *Cd14* ^+^ neutrophils. **(H)** The heatmap displaying the differential expression of genes in neutrophil clusters. **(I)** The schematic diagram displaying characteristics of *Cd14* ^+^ neutrophil in normal lung and *Ifit3* ^+^ neutrophil in breast cancer lung metastasis.

Similarly, we analyzed the subpopulations of neutrophils. Using the same parameters, neutrophils were divided into 10 clusters (**Supplementary Figure 4A**). The heatmap shows the top differentially expressed genes (**Supplementary Figure 4F**). And the violin plots show the fraction of mitochondria and ribosome genes (**Supplementary Figure 6G**). Through the difference analysis among clusters, we found four neutrophil subpopulations: *Ifi27l2a* ^+^ *Pim1* ^+^ *Cxcl2* ^+^ neutrophils, *Ppbp* ^+^ *Tsc22d1* ^+^ *Clec1b* ^+^ neutrophils, *Cd14* ^+^ *Vps37b* ^+^ *Eif4a1* ^+^ neutrophils, *Camp* ^+^ *Ngp* ^+^ *Ltf* ^+^ neutrophils (**Supplementary Figures 4B, C**). Moreover, there are great differences in the percentage of clusters across groups. There are higher proportions of cluster 5, 7, and 9 and lower proportions of cluster 1 in tumor-associated blood group than normal blood group (**Supplementary Figures 4D, E**). Two clusters have apparent differences in proportions between normal lung and lung metastasis groups (**Figures 4A, B**). Obviously, the percent of cluster 7 (*Ifit3* ^+^ neutrophils) is increased in the lung metastasis group and all metastatic stages. In the normal lung, the percent of cluster 8 (*Cd14* ^+^ neutrophils) is far higher than all lung-metastasis groups (**Figure 4C** and **Supplementary Figure 6C**). The hierarchical clustering displays the network of cluster classifications (**Figure 4D**).

Then, we compare differences in transcription between the two clusters. The dot plot shows that cluster 7 has the highest expression of *Ifit3* and cluster 8 has the highest expression of *Cd14* (**Figure 4E**). And the differentially expressed genes are shown in volcano plots, displaying the top-regulated genes of cluster 7 and 8 (**Figure 4F**). Next, we analyzed the GO biological process according to the differentially expressed genes (**Figure 4G**). Our results show that cluster 7 up-regulates some pathways involved in the viral process and response to interferon, and cluster 8 up-regulates many metabolic pathways. Furthermore, we found that *Isg15* and *Isg20*, encoding the secretory proteins which can promote tumor growth and metastasis (31, 32), are highly expressed in cluster 7. At last, we compared the expression of the pathway-associated genes among clusters, including *Ifit1*, *Ifit3*, *Ifit3b*, *Ifi47*, *Ifitm3*, and *Rsad2* of interferon-associated genes and *Vps37b*, *Eif4a1*, *Arg2*, *Dnaja1*, *Samd11*, and *Plk3* of metabolism-associated genes (**Figure 4H**) (33). The results indicate that higher expression of *Isg15*, *Isg20*, and interferon-associated genes appears in cluster 7 and higher expression of metabolism-associated genes appears in cluster 8. Our results show that *Ifit3* ^+^ neutrophils with high expression of *Isg15* and *Isg20* are associated with lung metastasis, and *Cd14* ^+^ neutrophils with high expression of metabolic genes are associated with normal lung (**Figure 4I**).

### *Il12b* ^+^ DCs are associated with lung metastasis through immunosuppression

**Figure 5:**
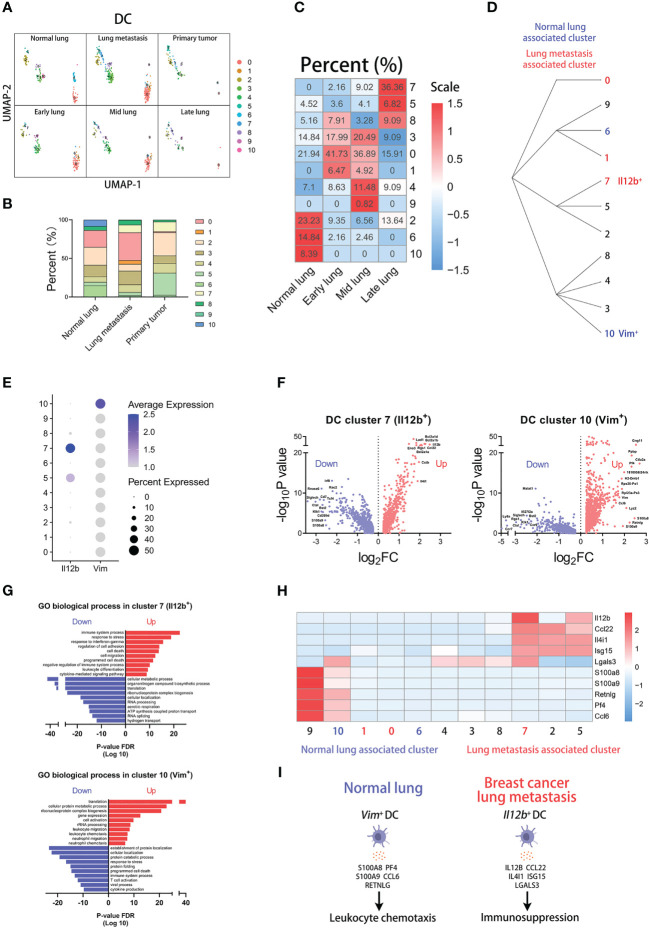
*Il12b* ^+^ DCs correlate with lung metastasis of breast cancer. **(A)** UMAP clustering of DCs in different tissues and lung metastasis stages. **(B)** The histogram comparing the percent of DC clusters in different tissues. **(C)** The heatmap displaying the percent of DC clusters in different lung metastasis stages. **(D)** Hierarchical clustering of DC clusters. **(E)** The dot plot of *Il12b* and *Vim* across DC clusters. **(F)** The volcano plots of differential gene expression of *Il12b* ^+^ and *Vim* ^+^ DCs. **(G)** The enrichment analysis of GO biological process in *Il12b* ^+^ and *Vim* ^+^ DCs. **(H)** The heatmap displaying the differential expression of genes in DC clusters. **(I)** The schematic diagram displaying characteristics of *Vim* ^+^ DC in normal lung and *Il12b* ^+^ DC in breast cancer lung metastasis.

For the exploration of DC subpopulations, DCs are divided into 11 clusters at the resolution of 1.8 (**Supplementary Figure 5A**). The heatmap displays the top marker genes of all clusters (**Supplementary Figure 5F**) and analysis of mitochondria and ribosome genes is shown in violin plots (**Supplementary Figure 6H**). Next, we identified three DC subpopulations: *Siglech* ^+^ *Ctsl* ^+^ *Klk1* ^+^ DCs, *Naaa* ^+^ *Ppt1* ^+^ *Plbd1* ^+^ DCs, *Ccl5* ^+^ *Ccr7* ^+^ *Fscn1* ^+^ DCs (**Supplementary Figures 5B, C**). In the subsequent analysis, we found that the group of normal blood comprises more proportions of cluster 9 and the group of tumor-associated blood comprises more proportions of cluster 5 (**Supplementary Figures 5D, E**). Furthermore, cluster 0, 1, and 7 have higher proportions in lung metastasis group than that in normal lung group, and cluster 2, 6, and 10 have higher proportions in normal lung group than that in lung metastasis group (**Figures 5A, B**). In particular, cluster 7 (*Il12b* ^+^ DCs) is not present in the normal lung and cluster 10 (*Vim* ^+^ DCs) is not present in the metastatic lung, which indicate that cluster 7 closely correlates with lung metastasis and cluster 10 closely correlates with normal lung (**Figure 5C** and **Supplementary Figure 6D**). The cluster classifications are shown by the hierarchical clustering (**Figure 5D**).

The dot plot shows that *Il12b* is mainly expressed in cluster 7 and *Vim* is mainly expressed in cluster 10 (**Figure 5E**). The differentially expressed genes of the two clusters are displayed by the volcano plots respectively (**Figure 5F**). To know which process and pathway are enriched in the regulated genes of the clusters, we performed the analysis of the GO biological process (**Figure 5G**). Interestingly, the results indicate that immune-associated processes are enriched in the up-regulated genes of both cluster 7 and 10. However, there are some differences in the enriched process of the two clusters. In the top up-regulated genes of two clusters, we found that some genes are associated with immunosuppression in cluster 7, including *Il12b*, *Ccl22*, *Il4i1*, *Isg15*, and *Lgals3* (34–37). Similarly, some genes are associated with leukocyte chemotaxis in cluster 10, including *S100a8*, *S100a9*, *Retnlg*, *Pf4*, and *Ccl6* (**Figure 5H**) (38–40). In conclusion, the above results indicate that *Il12b* ^+^ DCs with high expression of immunosuppression genes are associated with lung metastasis, and *Vim* ^+^ DCs with high expression of leukocyte chemotaxis genes are associated with normal lung (**Figure 5I**).

### Metastasis-associated myeloid cells are validated by MMTV-PyVT transgenic mice

**Figure 6:**
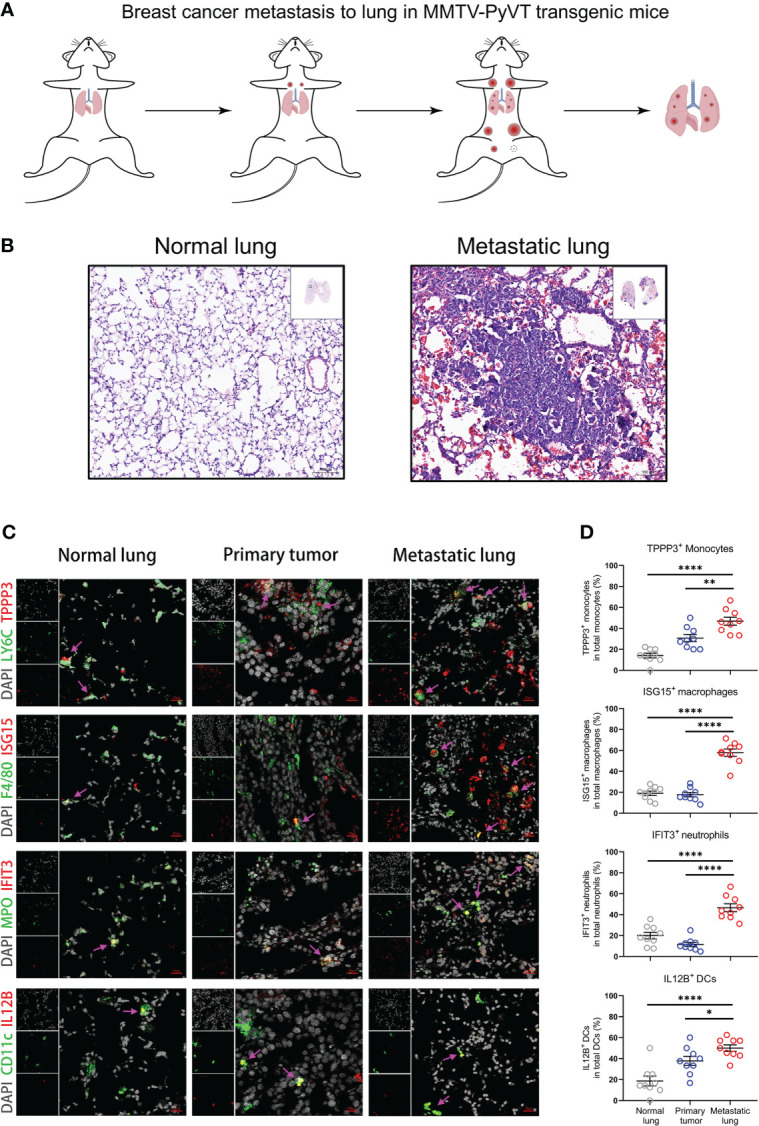
Metastasis-associated myeloid subpopulations are validated by MMTV-PyVT transgenic mice. **(A)** Schematic diagram of Breast cancer lung metastasis in MMTV-PyVT transgenic mice. **(B)** H&E staining images of lung from normal lung or metastatic lung of MMTV-PyVT mice. Scale bar, 100 μm. **(C, D)** The immunofluorescence shoots **(C)** showing metastasis-associated myeloid subpopulations in normal lung, primary tumor, and metastatic lung of MMTV-PyVT mice, which are summarized in **(D)** and each circle represents a field of immunofluorescence. Three fields of each biological replicate are used in analyses. Scale bar, 20 μm. Data are presented as the means ± SEM. *P < 0.05, **P < 0.01, ****P < 0.0001. The unpaired t-test in **(D)**.

To know whether our findings based on bioinformation analysis can be verified in mouse model, we examined the protein levels of the marker genes in metastasis-associated myeloid subpopulations. We employed the MMTV-PyVT transgenic mice, developing palpable mammary tumors which metastasize to the lung (11), to construct the mouse model of breast cancer lung metastasis (**Figure 6A**).

The lung tissues from MMTV-PyVT tumor-bearing mice are visibly infiltrated by tumor cells compared with the normal lung (**Figure 6B**). The tissue structure of the lung has been reconstructed by tumor infiltrates. Then, we performed immunofluorescence staining on the normal lung, primary tumor, and metastatic lung sections using the markers of *Tppp3* ^+^ monocytes (LY6C and TPPP3), *Isg15* ^+^ macrophages (F4/80 and ISG15), *Ifit3* ^+^ neutrophils (MPO and IFIT3), and *Il12b* ^+^ DCs (CD11c and IL12B) (**Figure 6C**). The metastasis-associated myeloid cells are indicated by pink arrows, which point out the colocalization of metastasis-associated markers and myeloid cells. The immunofluorescence results are summarized in the plots, which compare the proportions of metastasis-associated myeloid subpopulations between the normal lung or primary tumor and metastatic lung environments (**Figure 6D**). The results from MMTV-PyVT mice show that TPPP3^+^ monocytes, ISG15^+^ macrophages, IFIT3^+^ neutrophils, and IL12B^+^ DCs are mainly present in the tumor-reprogrammed lung microenvironment, and the metastasis-associated myeloid subpopulations are validated at the protein level. Moreover, there are some differences in proportions of myeloid subpopulations in primary tumor. Obviously, proportions of ISG15^+^ macrophages and IFIT3^+^ neutrophils in primary tumor have low levels as that in normal lung but not TPPP3^+^ monocytes and IL12B^+^ DCs, which indicate that these myeloid subpopulations may have different degrees of association with breast cancer metastasis to lung.

### Signatures of metastasis-associated myeloid cells correlate with poor progression of human breast cancer

**Figure 7:**
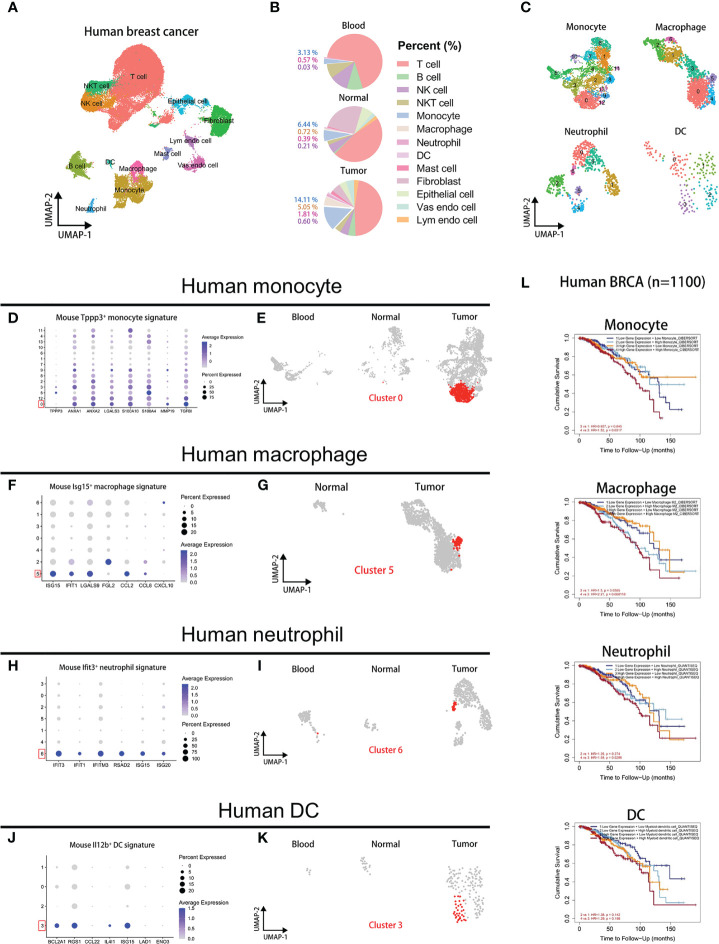
Signatures of metastasis-associated myeloid subpopulations are associated with poor progression of human breast cancer. **(A)** UMAP plot of 52562 annotated cells in GSE114727 of human breast cancer. **(B)** The pie plots displaying cell proportions of cell types in blood, normal, and tumor groups. **(C)** UMAP clustering of human monocyte, macrophage, neutrophil, and DC subsets. **(D)** The dot plot comparing signatures of mouse *Tppp3* ^+^ monocytes in human monocyte clusters. **(E)** UMAP plots labeled by cluster 0 of human monocytes. **(F)** The dot plot comparing signatures of mouse *Isg15* ^+^ macrophages in human macrophage clusters. **(G)** UMAP plots labeled by cluster 5 of human macrophages. **(H)** The dot plot comparing signatures of mouse *Ifit3* ^+^ neutrophils in human neutrophil clusters. **(I)** UMAP plots labeled by cluster 6 of human neutrophils. **(J)** The dot plot comparing signatures of mouse *Il12b* ^+^ DCs in human DC clusters. **(K)** UMAP plots labeled by cluster 3 of human DCs. **(L)** Survival plots showing associations clinical outcome and immune infiltrates combined with signature expression of the myeloid subpopulations in human BRCA (n=1100). High or low gene expression represents expression levels of marker genes of metastasis-associated myeloid populations.

Next, we sought to validate our findings of mouse myeloid cells in human data. However, there is no publicly available sc-RNAseq data of human associated with breast cancer metastasis to lung. So, we screened out a human breast cancer sc-RNAseq dataset (GSE114727) from the GEO database, containing monocytes, macrophages, neutrophils, and DCs. Through the analysis of the human sc-RNAseq data, we verified the signatures of mouse metastasis-associated myeloid cells in human. After the standard analysis workflow, we identified 13 subsets in human data: T cell, NKT cell, NK cell, B cell, neutrophil, monocyte, DC, macrophage, mast cell, epithelial cell, fibroblast, vascular endothelial cell, and lymphatic endothelial cell (**Figure 7A**). The distribution of all 13 populations in blood, normal, and tumor groups is shown in UMAP plots (**Supplementary Figure 7A**). And we calculated the proportions of cell types across tissues and myeloid cells (monocyte, macrophage, neutrophil, and DC) are mostly found in the tumor group (**Figure 7B**). To further perform alignment analysis between human and mouse myeloid cells, we separately reanalyzed the four myeloid subsets at the same parameters as follows: monocytes (14 clusters), macrophages (7 clusters), neutrophils (7 clusters), DCs (4 clusters) (**Figure 7C**).

To investigate whether our findings about mouse myeloid subsets are meaningful in human breast cancer data, we selected some marker genes of mouse myeloid cells and transformed them into human homologous genes. Firstly, we compared signatures of mouse normal lung subsets in human myeloid clusters, but we did not match the reliable clusters in human data for inconsistent and low levels of signatures (**Supplementary Figures 7B–E**). However, for mouse metastasis-associated myeloid subsets, we found that some human myeloid subsets have high expression of the mouse signatures and are mainly present in the tumor group. Human monocyte cluster 0 expressed the highest signatures of mouse *Tppp3* ^+^ monocytes and specially appeared in the tumor group (**Figures 7D, E**). Human macrophage cluster 5 highly expressed the most signatures of mouse *Isg15* ^+^ macrophages and specially appeared in the tumor group (**Figures 7F, G**). Human neutrophil cluster 6 highly expressed the all signatures of mouse *Ifit3* ^+^ neutrophils and specially appeared in the tumor group (**Figures 7H, I**). Human DC cluster 3 highly expressed the most signatures of mouse *Il12b* ^+^ DCs and specially appeared in the tumor group (**Figures 7J, K**). To investigate their associations with clinical outcome, we explored the clinical relevance of human breast invasive carcinoma (BRCA) between immune infiltrates and signature expression of metastasis-associated myeloid subpopulations by the TIMER2.0 (17). Signatures of four myeloid subpupolations are transformed into human homologous genes used in clinical outcome analysis. Representative human homologous signatures used in this analysis are as follows: *Tppp3* ^+^ Monocytes (*ANXA1*, *ANXA2*, *LGALS3*, *S100A10*, *S100A4*, *MMP19*, *TGFBI*), *Isg15* ^+^ macrophages (*ISG15*, *IFIT1*, *LGALS9*, *CCL2*, *CCL8*), *Ifit3* ^+^ neutrophils (*IFIT3*, *IFIT1*, *IFITM3*, *RSAD2*, *ISG15*, *ISG20*), *Il12b* ^+^ DCs (*BCL2A1*, *RGS1*, *IL4I1*, *ISG15*). The results show that patients with high signature expression and myeloid cell infiltrates observably have shorter survival (**Figure 7L**). These results indicate that mouse *Tppp3* ^+^ monocytes, *Isg15* ^+^ macrophages, *Ifit3* ^+^ neutrophils, and *Il12b* ^+^ DCs associated with lung metastasis of breast cancer can be partly validated in human data, and are predicted to closely correlate with poor tumor progression according to our analysis.

### Interaction analyses of mouse myeloid cells reveal a crosstalk network in metastatic lung

**Figure 8:**
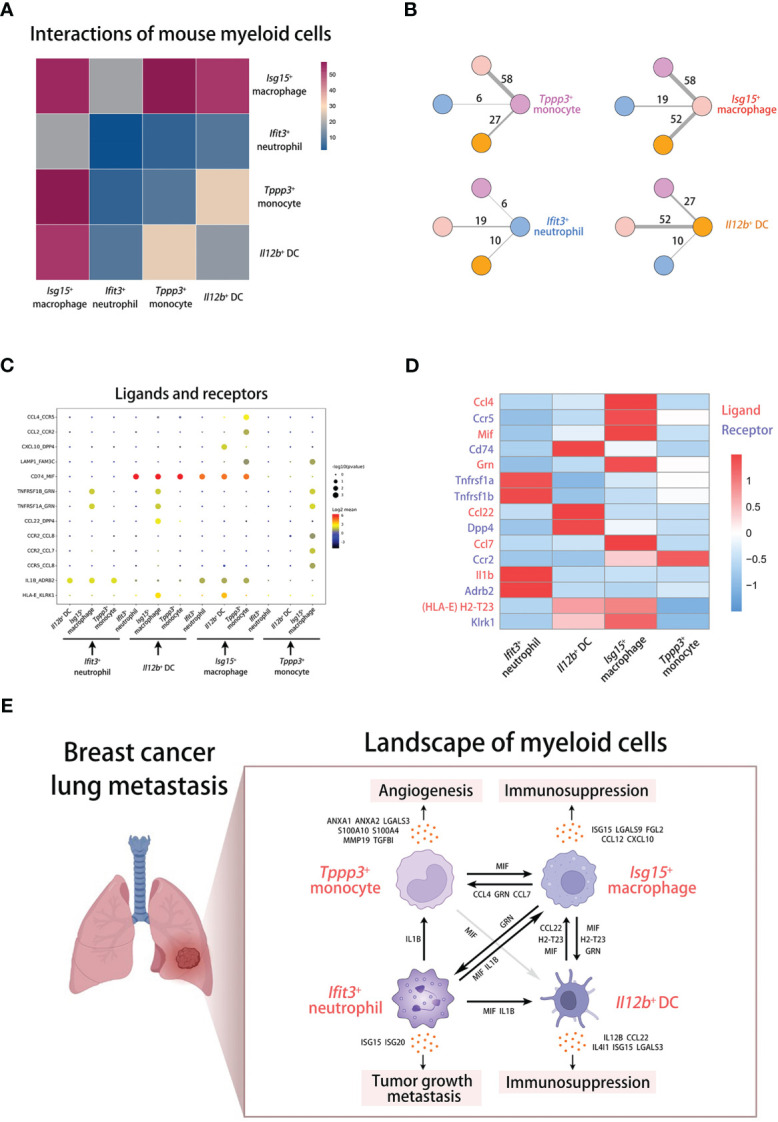
Cell-cell communications of myeloid cells in breast cancer lung metastasis. **(A)** The cell-cell communication analysis displaying interactions among mouse myeloid cells. **(B)** The shell plots of communication analysis among four mouse myeloid populations. **(C, D)** The dot plot **(C)** and heatmap **(D)** displaying interactions and gene expression of ligands and receptors across myeloid cells. **(E)** The schematic diagram displaying the landscape of myeloid cells in breast cancer lung metastasis.

Finally, we performed cell-cell communication analysis to examine the interactions among four metastasis-associated myeloid subsets by the “CellphoneDB” tool, a publicly available repository of curated receptors and ligands of human (18). To perform this analysis in mouse data, we transformed mouse genes into human homologous genes. The intensity of interactions is shown in the heatmap and the interaction values are displayed in the shell plots (**Figures 8A, B**). Blue to red bars represent the increase in intensity of the interactions of four myeloid cell populations. The pink, light red, bule, and yellow labels represent Tppp3+ monocytes, Isg15+ macrophages, Ifit3+ neutrophils, and Il12b+ DCs respectively. The results indicate that macrophages have the highest intensity of interactions with all other myeloid subsets.

To know the interactions in detail, we screened out some ligand-receptor groups from the analysis results and displayed them across four myeloid subsets in the dot plot (**Figure 8C**). For exploring the underlying mechanism of cell-cell communications, we focus on some prominent ligand-receptor groups, including CCL4-CCR5 (inflammation and cell chemotaxis), MIF-CD74 (immunoregulation and inflammation), GRN-TNFRSF1A/TNFRSF1B (inflammation and cell proliferation), CCL22-DPP4 (cell chemotaxis and immunoregulation), CCL7-CCR2 (cell chemotaxis), IL1B-ADRB2 (inflammation and immunoregulation), and HLA-E-KLRK1 (immunoregulation) (41–47). Then, we examined the expression of the matched mouse homologous genes in mouse myeloid subsets (**Figure 8D**). According to the analysis at the transcriptional level, *Isg15* ^+^ macrophages may participate in the chemotactic process of *Tppp3* ^+^ monocytes through CCL4 and CCL7, and regulate cell survival and activation through GRN. *Il12b* ^+^ DCs mainly mediate the chemotaxis of *Isg15* ^+^ macrophages through CCL22. Furthermore, *Ifit3* ^+^ neutrophils can activate other myeloid cells through IL1B. In particular, the transcript of MIF is highly expressed in all myeloid subsets. MIF derived from all cells including *Isg15* ^+^ macrophages can bind to CD74 on *Isg15* ^+^ macrophages. Interestingly, it has been reported that MIF can shape the heterogeneity of macrophages, which can induce pro-tumorigenic polarization of tumor-associated macrophages (48). The predicted mechanisms and cross-talk network of innate myeloid subpopulations in metastatic lung are shown in the scheme (**Figure 8E**).

## Discussion

We have identified four mouse myeloid subpopulations that highly correlate with breast cancer metastasis to lung, including *Tppp3* ^+^ monocytes, *Isg15* ^+^ macrophages, *Ifit3* ^+^ neutrophils, and *Il12b* ^+^ DCs. The pro-tumorigenic underlying mechanisms of them are elucidated and predicted according to differentially expressed genes and gene enrichment analysis. Furthermore, these myeloid subpopulations are validated in lung microenvironment of MMTV-PyVT transgenic mice, and their associations with poor progression of human breast cancer are uncovered by human RNAseq data from the TCGA. Also, they are partly verified in human sc-RNAseq data through the high expression of some signatures. And the matched myeloid clusters in human data are closely associated with tumor progression. In addition, we have examined and analyzed the interactions among metastasis-associated myeloid subpopulations. Altogether, our findings highlight the heterogeneity and specificity of tumor-associated myeloid cells and depict the interaction network of innate myeloid subpopulations in the tumor-reprogrammed lung microenvironment.

The critical role of myeloid cell populations has been recognized in the tumor microenvironment of both primary and secondary lung tumors (49). Myeloid-derived suppressor cells (MDSCs), heterogeneous cell populations suppressing T cell proliferation and cytokine production, are widely reported to have been involved in resistance to chemotherapy, targeted therapy, immunotherapy and prediction of poor prognosis (50). For instance, polymorphonuclear MDSCs (phenotypically resemble neutrophils) expressing IL-4R, IFN-γR, and Arg-1 are identified in treatment-naive advanced NSCLC patients and found inhibiting CD3ζ expression in CD8^+^ T lymphocytes (51). In our study, neutrophil cluster 3, 4 and 6 with high expression of *Il4ra*, *Ifngr1*, and *Arg1* also have a slight increase of proportions in lung metastases. Neutrophil cluster 6 also has high expression of *Ccl2*, *Ccl3*, *Ccl4*, *Ccl5*, and *Cxcl2*, which are inflammatory mediators involved in MDSC migration to and activation in the tumor microenvironment (52). Interestingly, a study reported that monocytic MDSCs expressing high levels of the proinflammatory molecule S100A8 and S100A9 are highly correlated with the ability to suppress T cell proliferation (53). However, monocyte cluster 8 with high expression of *S100a8* and *S100a9* found in our study is less present in lung metastases and primary tumor. Moreover, DC cluster 0 and 1 more present in lung metastases highly expressed *Tgfb1*, encoding TGFβ which induces the differentiation of CD4^+^ T cells into CD4^+^CD25^+^FOXP3^+^ regulatory T cells that suppress T cell proliferation (54). In addition, macrophage cluster 3, 8, and 10 associated with tumor progression or metastasis express low levels of *Mrc1* (CD206) and high levels of *Vegfa*, which indicate a phenotype of M2d macrophages facilitating tumor angiogenesis (55).

Recent studies have dissected the heterogeneity of myeloid cells in lung cancer by single-cell technology. For instance, analysis of single-cell transcriptomics mapped tumor-infiltrating myeloid cells and uncovered the conserved myeloid populations in human and mouse lung cancers (56). However, the differences in myeloid populations between normal lung and lung cancer have not been explored. In particular, two subpopulations identified as metastasis-associated subsets in our study are also found in the research previously, including *Ifit3* ^+^ *Ifit1* ^+^ *Irf7* ^+^ *Rsad2* ^+^ neutrophils and *Il12b* ^+^ *Ccl22* ^+^ DCs. But the impact and mechanism of promoting tumor progression have not been assessed. Another research described the diversity within the myeloid cell lineage and functionality by single-cell RNA sequencing of metastatic lung adenocarcinoma (57). And *MIF* ^+^ *CXCL3* ^+^ *CCL20* ^+^ macrophages and *ILRB4* ^+^ *LILRA4* ^+^ *GZMB* ^+^ plasmacytoid DCs are mainly detected in the metastases and considered priming suppressive immune microenvironment. Moreover, the profibrotic and immunosuppressive monocyte/macrophage population (CD14^+^CD16^+^CD81^+^) was identified in SCLC (small cell lung cancer) tumors, related to PLCG2-high SCLC phenotype with stem-like and pro-metastatic features (58). Tumor-associated myeloid cells of innate immune system have emerged as the key role of lung cancer progression.

Our study defined four metastasis-associated myeloid populations and predicted the pro-tumorigenic molecular mechanisms through some secretory factors. The angiogenic function of *Tppp3* ^+^ monocyte is mainly supported by high expression of *Anxa1* and *Anxa2*, encoding proteins involved in tumor angiogenesis and metastasis (19, 59). And this population has not been reported and investigated previously. For *Isg15*-expressing macrophages, it was reported that ISG15 in macrophages participates in a complex regulatory network governing cell respiration and metabolism (60). Also, ISG15 has been reported to promote tumor cell migration and was considered to induce the immunosuppressive phenotype of macrophages (61). Here, we investigated the key role of *Isg15* ^+^ macrophages in lung metastasis through the high expression of immunosuppressive genes encoding secretory proteins. In addition, our analysis identified an IFN-responsive neutrophil population with high expression of IFN-responsive genes (*Ifit3*, *Ifit1*, *Ifit3b*, *Ifi47*, *Ifitm3*, *Rsad2*, *Isg15*, and *Isg20*), which is more present in metastatic lung of breast cancer. These factors can be induced by interferon signaling derived from T cells and NK cells (33, 62). And the *Ifit3* ^+^ neutrophils may have strong interactions with lymphoid cells, which has not been explored in this study and should be paid more attention. Particularly, two interferon-stimulated factors (ISG15 and ISG20) are also found contributing to tumor growth and metastasis (31, 32). *Il12b*-expressing DCs identified in this study are found relating to lung metastasis and IL12B can associate with IL23A to form the IL-23 interleukin, which leads to the generation of IL-17-producing CD4^+^ T cells and is important for tumorigenesis (34). Another dominant immunosuppressive gene (*Ccl22*) is also highly expressed in *Il12b* ^+^ DCs, which is identified in lung metastases. The encoded chemokine CCL22 mediates the trafficking of regulatory T cells to the tumor, by which tumors may foster immune privilege (35). Except for the above myeloid populations, there are other populations identified in normal lung, however, we have not verified their signatures in human data and these populations may need to be examined and validated in the larger datasets. And other clusters not deeply investigated in our study have also the potential to participate in the metastatic process, including monocyte cluster 4 and 7, macrophage cluster 1 and 10, and DC cluster 0 and 1. In addition, there are some limitations of the study, which should be performed more research in the future. For instance, the expression of transcriptional genes should be verified at the protein levels, and the pro-metastasis function of myeloid subpopulations needs to be assessed and investigated *in vivo* through some experimental techniques.

Our studies explore the heterogeneity and specificity of metastasis-associated myeloid cells and describe the landscape of innate myeloid populations in tumor-reprogrammed lung microenvironment. The exploration and description contribute to the establishment of the crosstalk network of innate myeloid cells in lung metastasis, which can facilitate the research about lung-metastasis mechanisms and provide new insights for multi-target therapeutic strategies of breast cancer metastasis to lung.

## Data availability statement

Publicly available datasets were analyzed in this study. This data can be found here: https://www.ncbi.nlm.nih.gov/geo/query/acc.cgi?acc=GSE109281, GSE109281, GEO, NCBI; https://www.ncbi.nlm.nih.gov/geo/query/acc.cgi?acc=GSE129578, GSE129578, GEO, NCBI; https://www.ncbi.nlm.nih.gov/geo/query/acc.cgi?acc=GSE114727, GSE114727, GEO, NCBI.

## Ethics statement

The animal study was reviewed and approved by Laboratory Animal Ethics Committee of West China Second University Hospital, Sichuan University.

## Author contributions

ZH collected and analyzed the data, performed the experiments, and wrote the manuscript. DB and NY performed the experiments. WH, LZ, and XL provided suggestions on the project design. B-SD conceived and supervised the project, and revised the manuscript. All authors contributed to the article and approved the submitted version.
