# Supplementary figures and images for: Integrated analyses of single-cell transcriptomics identify metastasis-associated myeloid subpopulations in breast cancer lung metastasis
Source: Front Immunol. 2023 Jul 7;14:1180402. doi: 10.3389/fimmu.2023.1180402 (PMC10361816; doi:10.3389/fimmu.2023.1180402)

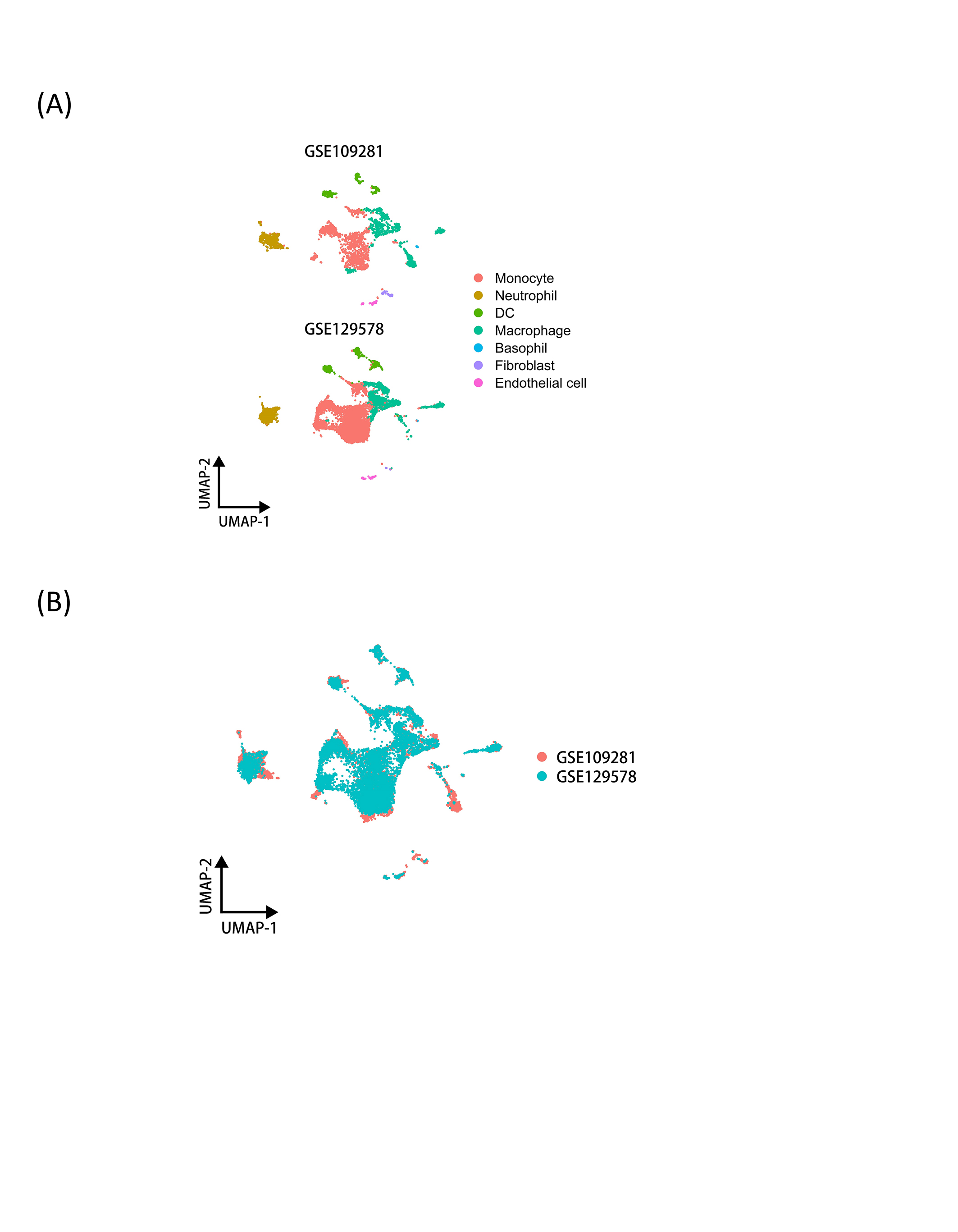

Supplement: Supplementary file 1 [file Image_1.jpeg]

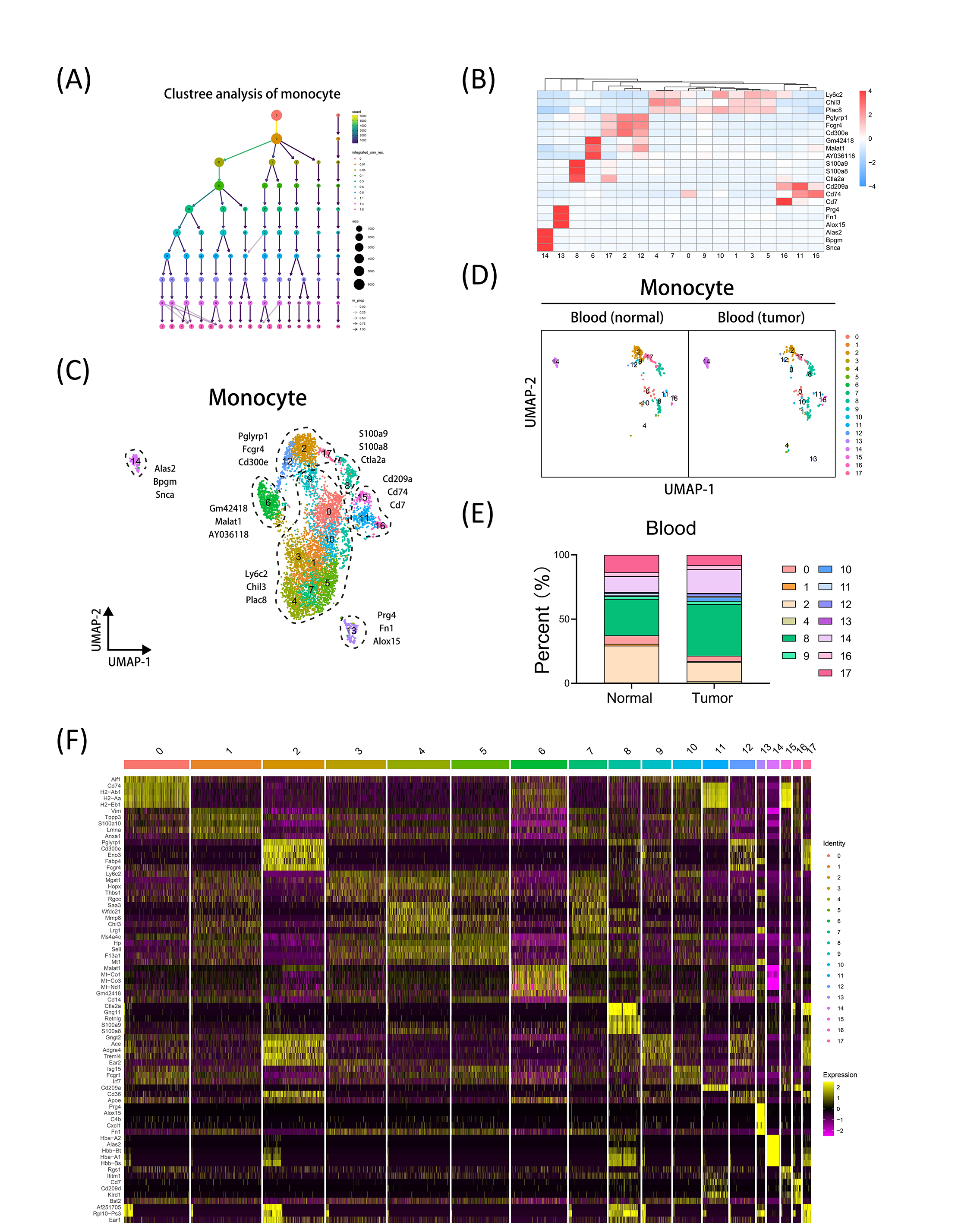

Supplement: Supplementary file 2 [file Image_2.jpeg]

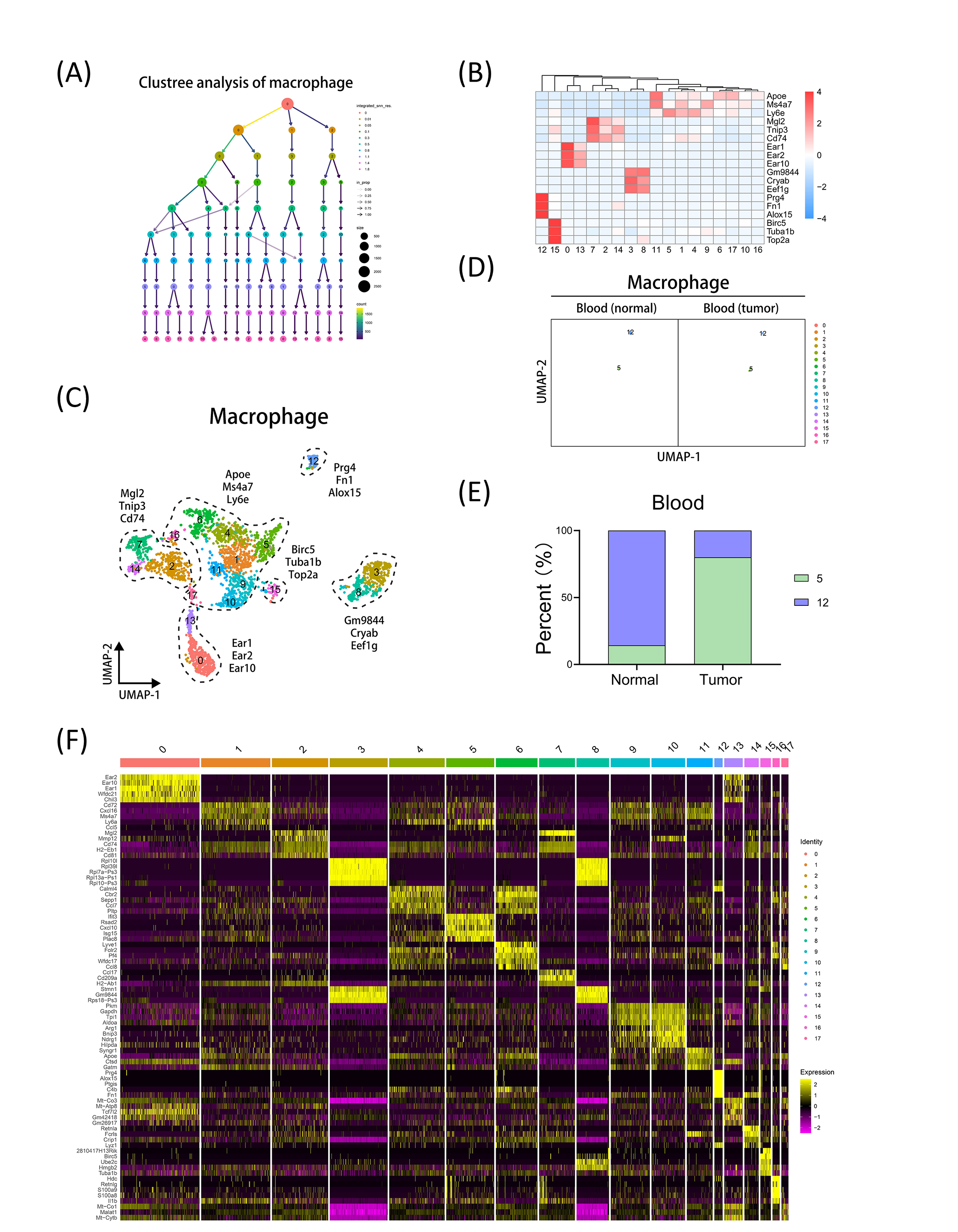

Supplement: Supplementary file 3 [file Image_3.jpeg]

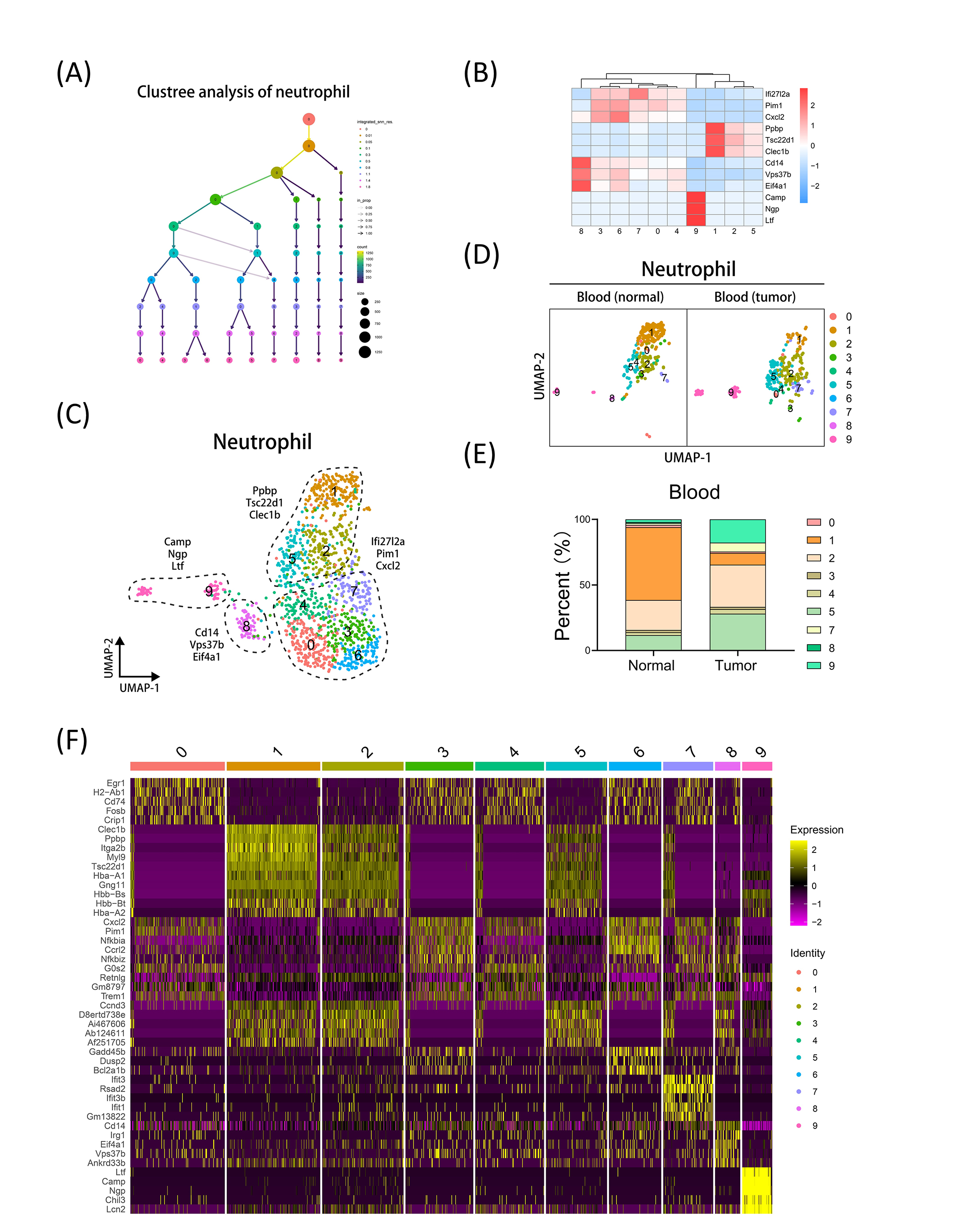

Supplement: Supplementary file 4 [file Image_4.jpeg]

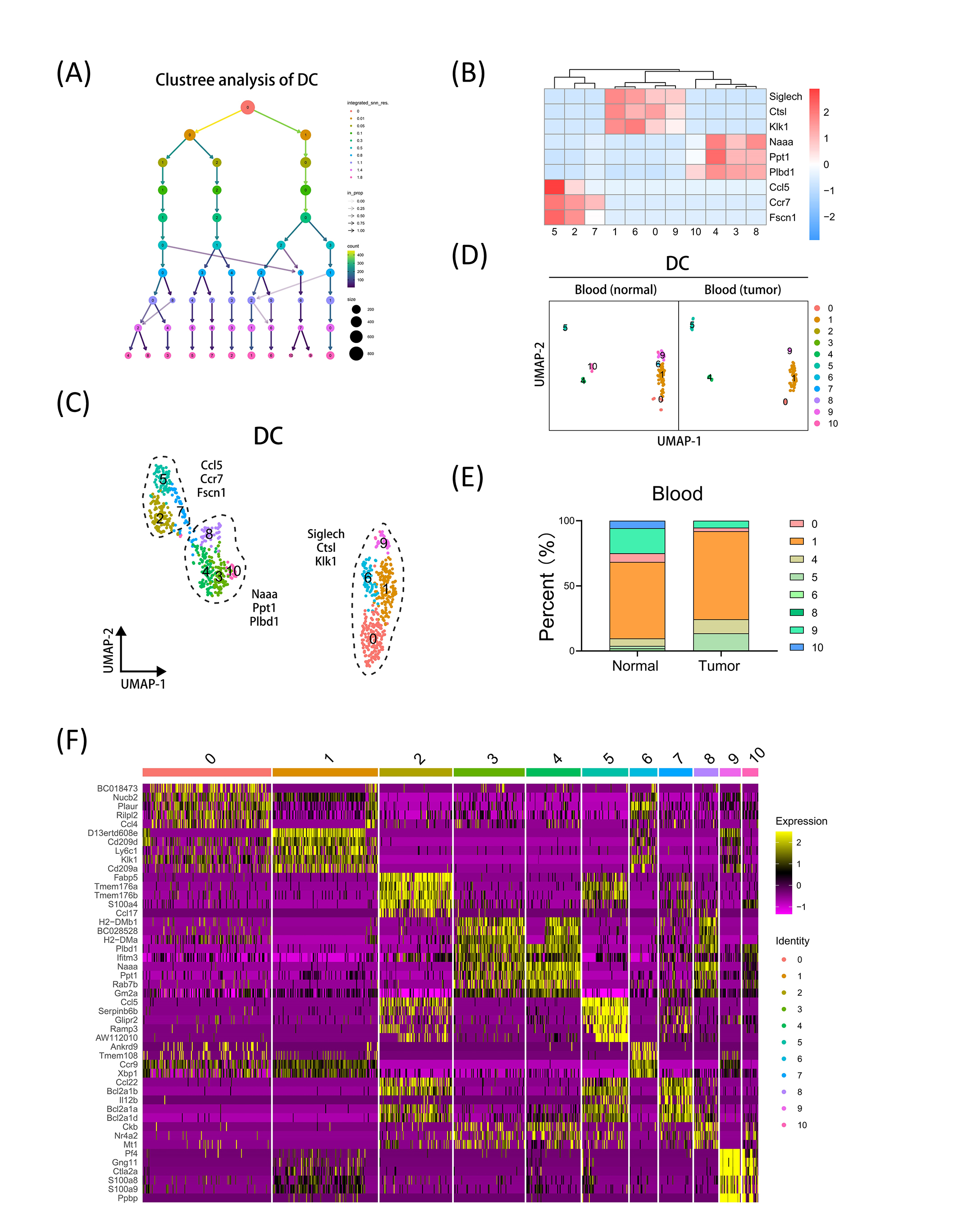

Supplement: Supplementary file 5 [file Image_5.jpeg]

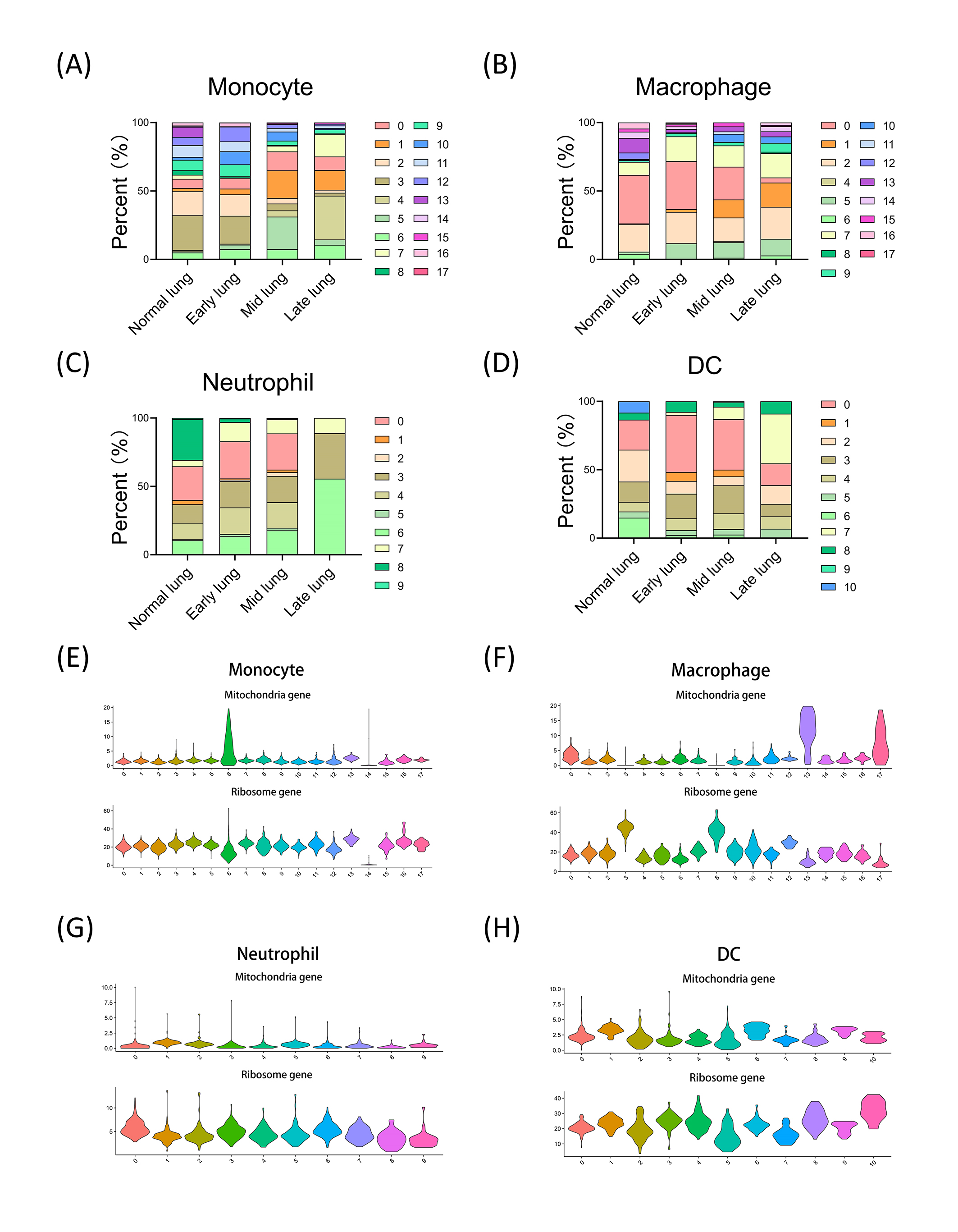

Supplement: Supplementary file 6 [file Image_6.jpeg]

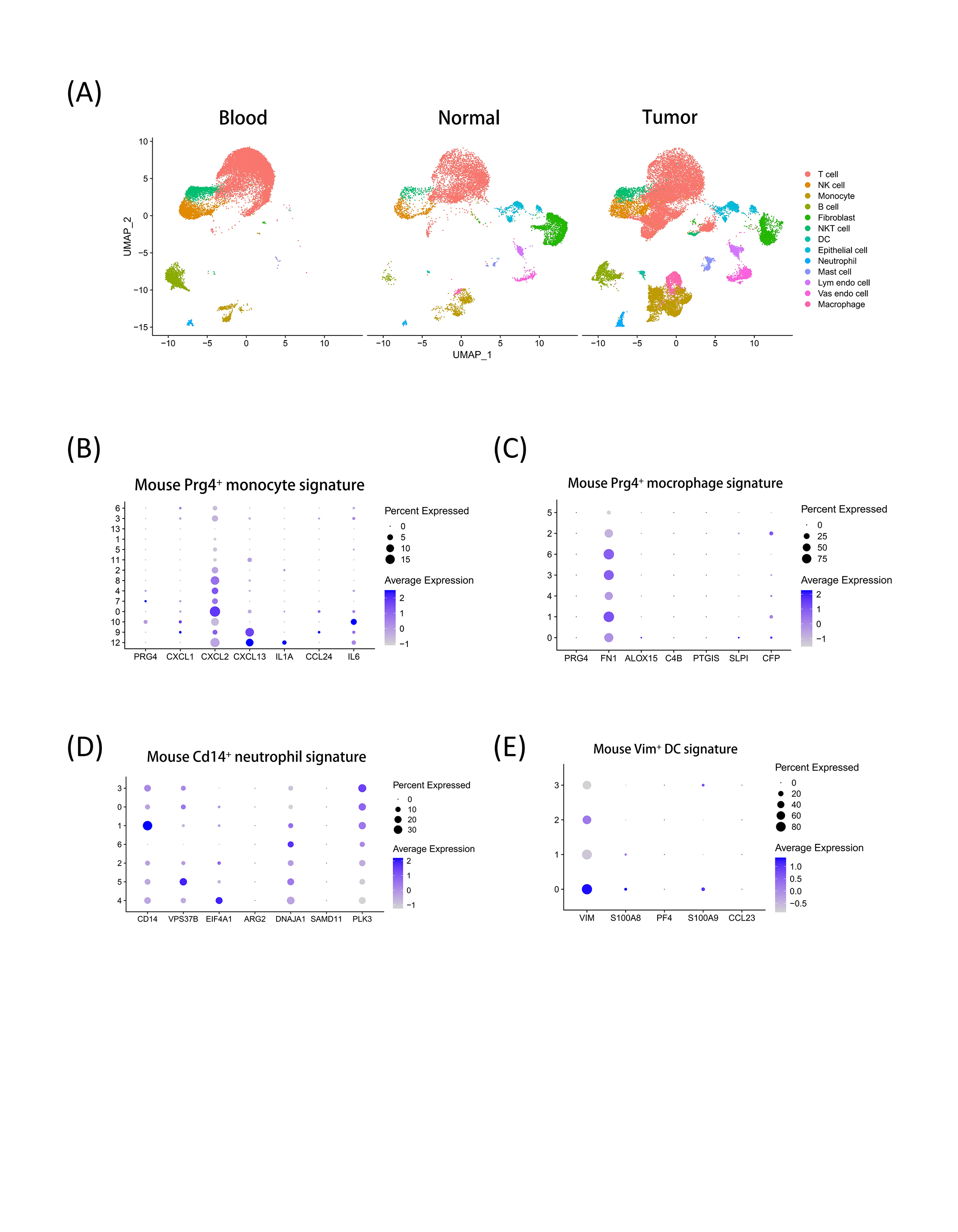

Supplement: Supplementary file 7 [file Image_7.jpeg]
